# Dichlorido{2,6-diisopropyl-*N*-[(*S*)-pyrrolidin-2-ylmeth­yl]aniline-κ^2^ *N*,*N*′}palladium(II)

**DOI:** 10.1107/S1600536813008271

**Published:** 2013-04-05

**Authors:** Saira Nayab, Hong-In Lee, Jong Hwa Jeong

**Affiliations:** a Department of Chemistry, Kyungpook National University, Taegu 702-701, Republic of Korea

## Abstract

In the title compound, [PdCl_2_(C_17_H_28_N_2_)], the Pd^II^ atom displays a square-planar coordination involving two N atoms of a 2,6-diisopropyl-*N*-[(*S*)-pyrrolidin-2-ylmeth­yl]aniline ligand and two chloride ligands, with a deviation of 0.090 (1) Å for the Pd^II^ atom from the best plane. The absolute configuration of the chiral C atom of the pyrrolidine ring is *S*, which induces *R* configurations at the two N atoms of the aniline ligand. Optical isomerism arising from the chelate five-membered ring is configured as δ. The Pd—N bond lengths are 2.040 (3) and 2.072 (2) Å, and the Pd—Cl bond lengths are 2.3055 (8) and 2.3160 (8) Å. In the crystal, pairs of N—H⋯Cl hydrogen bonds link mol­ecules into discrete dimers.

## Related literature

For background to the use of palladium complexes bearing enanti­opure ligands in asymmetric synthesis, see: Sodeoka & Hamashima (2006 ▶); Quintard *et al.* (2008 ▶); Tan *et al.* (2009 ▶) and as anti­cancer drugs, see: Barnham *et al.* (1994 ▶). For the synthesis of the 2,6-diisopropyl-*N*-[(*S*)-pyrrolidin-2-ylmeth­yl]aniline ligand, see: Shifeng *et al.* (2010 ▶). For related structures, see: Rafii *et al.* (2007 ▶). For a description of the Cambridge Structural Database, see: Allen *et al.* (2002 ▶).
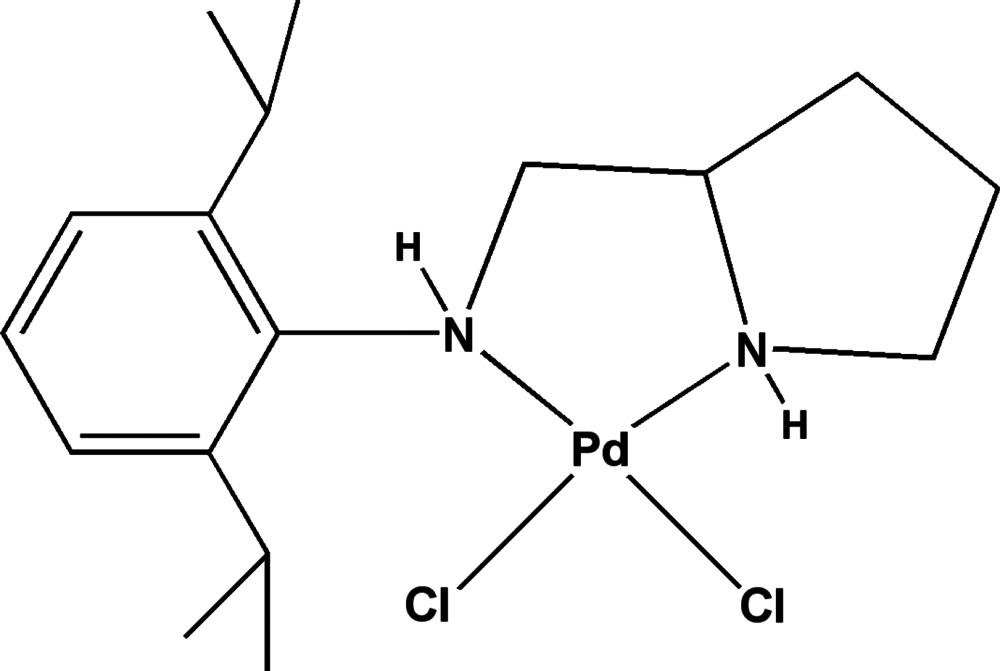


## Experimental

### 

#### Crystal data

[PdCl_2_(C_17_H_28_N_2_)]
*M* *_r_* = 437.71
Monoclinic, 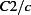

*a* = 24.287 (3) Å
*b* = 8.6534 (12) Å
*c* = 18.355 (2) Å
β = 94.851 (9)°
*V* = 3843.7 (8) Å^3^
*Z* = 8
Mo *K*α radiation
μ = 1.24 mm^−1^
*T* = 293 K
0.45 × 0.40 × 0.40 mm

#### Data collection

Enraf–Nonius CAD-4 four-circle diffractometer
Absorption correction: ψ scan (*ABSCALC*; McArdle & Daly, 1999 ▶) *T*_min_ = 0.578, *T*_max_ = 0.608
3790 measured reflections
3580 independent reflections
3089 reflections with *I* > 2σ(*I*)
*R*_int_ = 0.018
3 standard reflections every 60 min intensity decay: 0.2%

#### Refinement

*R*[*F* ^2^ > 2σ(*F* ^2^)] = 0.038
*wR*(*F* ^2^) = 0.105
*S* = 1.08
3580 reflections
199 parameters
H-atom parameters constrained
Δρ_max_ = 1.33 e Å^−3^
Δρ_min_ = −1.56 e Å^−3^

### 

Data collection: *CAD-4 Software* (Enraf–Nonius, 1989 ▶); cell refinement: *CAD-4 Software*; data reduction: *XCAD* (McArdle, 1999 ▶); program(s) used to solve structure: *SHELXS97* (Sheldrick, 2008 ▶); program(s) used to refine structure: *SHELXL97* (Sheldrick, 2008 ▶); molecular graphics: *ORTEPIII* (Burnett & Johnson, 1996 ▶); software used to prepare material for publication: *SHELXL97*.

## Supplementary Material

Crystal structure: contains datablock(s) global, I. DOI: 10.1107/S1600536813008271/fk2069sup1.cif

Structure factors: contains datablock(s) I. DOI: 10.1107/S1600536813008271/fk2069Isup2.hkl

Additional supplementary materials:  crystallographic information; 3D view; checkCIF report

## Figures and Tables

**Table 1:** Hydrogen-bond geometry (Å, °)

*D*—H⋯*A*	*D*—H	H⋯*A*	*D*⋯*A*	*D*—H⋯*A*
N1—H1⋯Cl2^i^	0.86	2.47	3.283 (3)	158
N2—H2⋯Cl1^i^	0.86	2.68	3.410 (3)	144

Symmetry code: (i) 
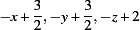
.

**Fig. 1.**
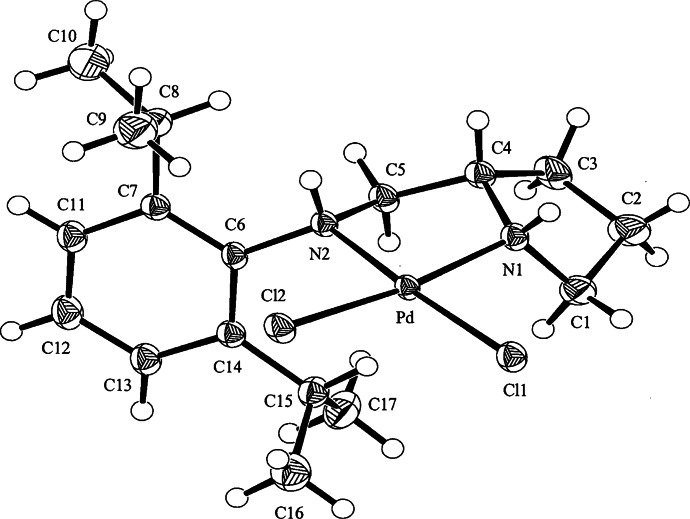
The molecular structure of the title compound. Displacement ellipsoids are drawn at the 40% probability level.

**Fig. 2.**
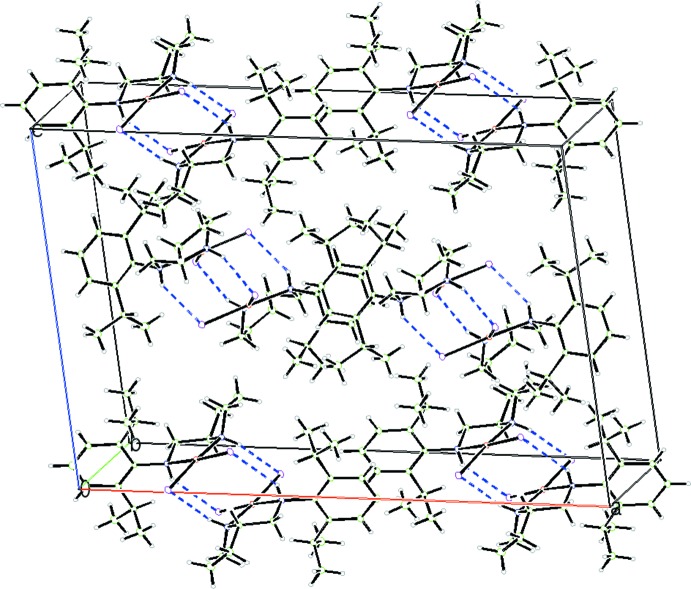
Packing diagram of the title compound approximately viewed along b-axis. Hydrogen bonds are indicated by dashed lines.

## supplementary crystallographic information

### Comment

Palladium complexes of various types bearing the enantiopure ligands are widely used in modern asymmetric synthesis (Sodeoka *et al.*, 2006). Palladium complexes (Quintard *et al.*, 2008; Tan *et al.*, 2009) containing homochiral diamine ligands derivable from natural amino acids are now well established in the clinical treatment as anticancer drugs (Barnham *et al.*, 1994). In this paper, we describe synthesis and the crystal structure of novel chiral dichloro Pd(II) complex bearing the ligand 2,6-diisopropyl-N-(*S*-pyrrolidin-2-yl) methyl)benzenamine which was prepared by the reported method (Shifeng *et al.*, 2010). The geometry around the Pd(II) centre is almost square-planar (Fig. 1). The coordination plane composed of Pd, Cl1, Cl2, N1, and N2 is nearly coplanar within 0.090 (1) Å deviation from the best plane. The bite angle of N1—Pd—N2 [84.0 (1) °] is much smaller than the Cl1—Pd—Cl2 [93.10 (3) °] angle. The chiral C atom of the pyrrolidine moiety has *S* configuration and the induced chiralities at two N atoms of the ligand show *R* configuration. The orientation of the hydrogen atoms of the chiral C and N atoms is in head-to-head. Optical isomerism arising from the chelate five-membered ring is configured as δ. The bond lengths of Pd—N are 2.040 (3) and 2.072 (2) Å and those of Pd—Cl are 2.3055 (8) and 2.3160 (8) Å. These bond lengths are similar to the known average Pd—N and Pd—Cl lengths of (1*R*,2*R*)-(1,2-bisbenzyl)-1,2- diaminocyclohexane palladium dichloride complex (Rafii *et al.*, 2007). There are two intermolecular N—H···Cl between each two molecules to form discrete dimers as shown in Fig. 2. Hydrogen-bond parameters are listed in Table 1.

### Experimental

The ligand, 2,6-diisopropyl-*N*-(*S*-pyrrolidin-2-yl)methyl) benzenamine, was prepared by the reported method (Shifeng *et al.*, 2010). Ligand (0.30 g, 1.15 mmol) solution in CH_3_CN (7 ml) was treated with PdCl_2_(CH_3_CN)_2_ (0.30 g, 1.15 mmol) in CH_3_CN (10 ml) at ambient temperature for overnight. The solvent was removed under reduced pressure to get brown orange reside. Washing the precipitate with cold Et_2_O afforded orange solid as the final product (0.38 g, 76%). Anal. Calcd. for C_17_H_28_Cl_2_N_2_Pd: C, 46.64; H, 6.45; N, 6.40. Found: C, 46.60; H, 6.51; N, 6.37%. ^1^H NMR (400 MHz, CDCl_3_) δ 7.19 (m, 2H, Ar*H*), 7.05 [m, 1H, Ar*H*], 3.73 (m, 2H, PyC*H*_2_), 3.52 (m, 2H, ArC*H*_2_), 3.32 (m, 2H, py*H* & ArN*H*), 3.00–2.64 (m, 2H, py*H*), 2.60- 2.51 (br s, 1H, PyN*H*), 1.91 (m, 2H, py*H*), 1.63–1.57 (m, 2H, py*H*), 1.29 (*d*, J = 6.8 Hz, 12H, 4 C*H*_3_).

### Refinement

All H atoms were positioned geometrically and refined using a riding model with C—H 0.93 - 0.98 Å, N—H 0.86 Å and *U*_iso_ = 1.5*U*_eq_(C) for CH_3 and_ 1.2*U*_eq_(C,N).

### Crystal data

**Table:**

[PdCl_2_(C_17_H_28_N_2_)]	*F*(000) = 1792
*M**_r_* = 437.71	*D*_x_ = 1.513 Mg m^−^^3^
Monoclinic, *C*2/*c*	Mo *K*α radiation, λ = 0.71073 Å
Hall symbol: -C 2yc	Cell parameters from 25 reflections
*a* = 24.287 (3) Å	θ = 9.0–13.0°
*b* = 8.6534 (12) Å	µ = 1.24 mm^−^^1^
*c* = 18.355 (2) Å	*T* = 293 K
β = 94.851 (9)°	Brick, orange
*V* = 3843.7 (8) Å^3^	0.45 × 0.40 × 0.40 mm
*Z* = 8	

### Data collection

**Table:**

Enraf–Nonius CAD-4 four-circle diffractometer	3089 reflections with *I* > 2σ(*I*)
Radiation source: fine-focus sealed tube	*R*_int_ = 0.018
Graphite monochromator	θ_max_ = 25.5°, θ_min_ = 1.7°
ω/2θ scans	*h* = 0→29
Absorption correction: ψ scan (ABSCALC; McArdle & Daly, 1999)	*k* = −10→0
*T*_min_ = 0.578, *T*_max_ = 0.608	*l* = −22→22
3790 measured reflections	3 standard reflections every 60 min
3580 independent reflections	intensity decay: 0.2%

### Refinement

**Table:**

Refinement on *F*^2^	Primary atom site location: structure-invariant direct methods
Least-squares matrix: full	Secondary atom site location: difference Fourier map
*R*[*F*^2^ > 2σ(*F*^2^)] = 0.038	Hydrogen site location: inferred from neighbouring sites
*wR*(*F*^2^) = 0.105	H-atom parameters constrained
*S* = 1.08	*w* = 1/[σ^2^(*F*_o_^2^) + (0.0819*P*)^2^ + 0.2845*P*] where *P* = (*F*_o_^2^ + 2*F*_c_^2^)/3
3580 reflections	(Δ/σ)_max_ = 0.004
199 parameters	Δρ_max_ = 1.33 e Å^−^^3^
0 restraints	Δρ_min_ = −1.56 e Å^−^^3^

### Special details

**Table:**

Geometry. All e.s.d.'s (except the e.s.d. in the dihedral angle between two l.s. planes) are estimated using the full covariance matrix. The cell e.s.d.'s are taken into account individually in the estimation of e.s.d.'s in distances, angles and torsion angles; correlations between e.s.d.'s in cell parameters are only used when they are defined by crystal symmetry. An approximate (isotropic) treatment of cell e.s.d.'s is used for estimating e.s.d.'s involving l.s. planes.

### Fractional atomic coordinates and isotropic or equivalent isotropic displacement parameters (Å^2^)

**Table:**

	*x*	*y*	*z*	*U*_iso_*/*U*_eq_	
Pd	0.792743 (8)	0.79819 (2)	0.934349 (11)	0.02593 (12)	
Cl1	0.71265 (3)	0.92407 (9)	0.89218 (4)	0.0389 (2)	
Cl2	0.83021 (3)	1.01710 (9)	0.99173 (5)	0.0391 (2)	
N1	0.76098 (10)	0.5922 (3)	0.89652 (14)	0.0320 (5)	
H1	0.7308	0.5786	0.9169	0.038*	
N2	0.86249 (10)	0.6681 (3)	0.96516 (14)	0.0296 (5)	
H2	0.8597	0.6456	1.0103	0.035*	
C1	0.74724 (15)	0.5713 (4)	0.81664 (19)	0.0448 (8)	
H1A	0.7770	0.6075	0.7889	0.054*	
H1B	0.7135	0.6253	0.8001	0.054*	
C2	0.74001 (19)	0.3971 (4)	0.8098 (2)	0.0560 (10)	
H2A	0.7043	0.3650	0.8245	0.067*	
H2B	0.7436	0.3629	0.7601	0.067*	
C3	0.78631 (16)	0.3348 (4)	0.8613 (2)	0.0518 (10)	
H3A	0.8189	0.3156	0.8356	0.062*	
H3B	0.7754	0.2388	0.8833	0.062*	
C4	0.79815 (12)	0.4599 (4)	0.92021 (18)	0.0352 (7)	
H4	0.7888	0.4211	0.9678	0.042*	
C5	0.85712 (12)	0.5168 (3)	0.92571 (19)	0.0362 (7)	
H5A	0.8808	0.4409	0.9515	0.043*	
H5B	0.8692	0.5287	0.8770	0.043*	
C6	0.91821 (11)	0.7313 (3)	0.96309 (17)	0.0291 (6)	
C7	0.95576 (13)	0.7160 (3)	1.02479 (18)	0.0327 (7)	
C8	0.93964 (13)	0.6498 (4)	1.09665 (17)	0.0372 (7)	
H8	0.9095	0.5762	1.0852	0.045*	
C9	0.9180 (2)	0.7757 (5)	1.1435 (3)	0.0637 (12)	
H9A	0.9469	0.8491	1.1562	0.096*	
H9B	0.9058	0.7311	1.1873	0.096*	
H9C	0.8876	0.8270	1.1169	0.096*	
C10	0.98669 (18)	0.5623 (6)	1.1401 (2)	0.0643 (11)	
H10A	1.0162	0.6329	1.1544	0.096*	
H10B	1.0002	0.4822	1.1101	0.096*	
H10C	0.9732	0.5173	1.1829	0.096*	
C11	1.00930 (14)	0.7704 (4)	1.0203 (2)	0.0406 (8)	
H11	1.0347	0.7629	1.0610	0.049*	
C12	1.02549 (14)	0.8350 (5)	0.9571 (2)	0.0463 (8)	
H12	1.0616	0.8691	0.9548	0.056*	
C13	0.98797 (13)	0.8487 (4)	0.8975 (2)	0.0410 (7)	
H13	0.9993	0.8927	0.8550	0.049*	
C14	0.93362 (13)	0.7992 (3)	0.89800 (18)	0.0319 (7)	
C15	0.89514 (13)	0.8238 (4)	0.82901 (18)	0.0383 (7)	
H15	0.8582	0.7891	0.8393	0.046*	
C16	0.89141 (18)	0.9966 (5)	0.8104 (3)	0.0623 (11)	
H16A	0.9274	1.0348	0.8020	0.093*	
H16B	0.8776	1.0520	0.8504	0.093*	
H16C	0.8668	1.0113	0.7672	0.093*	
C17	0.91266 (18)	0.7291 (6)	0.7649 (2)	0.0580 (11)	
H17A	0.9496	0.7572	0.7552	0.087*	
H17B	0.8879	0.7492	0.7224	0.087*	
H17C	0.9116	0.6211	0.7768	0.087*	

### Atomic displacement parameters (Å^2^)

**Table:**

	*U*^11^	*U*^22^	*U*^33^	*U*^12^	*U*^13^	*U*^23^
Pd	0.02146 (17)	0.02124 (17)	0.03568 (17)	0.00033 (7)	0.00598 (10)	−0.00046 (8)
Cl1	0.0303 (4)	0.0360 (4)	0.0503 (5)	0.0077 (3)	0.0037 (3)	0.0045 (3)
Cl2	0.0330 (4)	0.0265 (4)	0.0583 (5)	−0.0053 (3)	0.0064 (3)	−0.0055 (3)
N1	0.0259 (12)	0.0289 (13)	0.0417 (14)	−0.0020 (10)	0.0056 (10)	−0.0027 (11)
N2	0.0253 (12)	0.0252 (11)	0.0385 (14)	0.0003 (10)	0.0048 (10)	0.0039 (11)
C1	0.054 (2)	0.0314 (17)	0.0469 (19)	0.0011 (14)	−0.0072 (15)	−0.0049 (14)
C2	0.077 (3)	0.0322 (18)	0.057 (2)	−0.0009 (18)	−0.0068 (19)	−0.0135 (17)
C3	0.053 (2)	0.0223 (15)	0.079 (3)	−0.0006 (15)	−0.0031 (19)	−0.0053 (17)
C4	0.0348 (15)	0.0246 (15)	0.0461 (17)	−0.0013 (12)	0.0030 (13)	0.0054 (13)
C5	0.0317 (15)	0.0245 (15)	0.0526 (19)	0.0015 (12)	0.0054 (13)	−0.0026 (13)
C6	0.0207 (13)	0.0271 (14)	0.0399 (16)	0.0026 (11)	0.0048 (11)	−0.0020 (12)
C7	0.0309 (15)	0.0262 (15)	0.0409 (17)	0.0031 (11)	0.0027 (13)	−0.0018 (12)
C8	0.0385 (17)	0.0371 (17)	0.0354 (16)	−0.0002 (14)	−0.0005 (13)	0.0023 (14)
C9	0.075 (3)	0.057 (3)	0.063 (3)	0.006 (2)	0.029 (2)	−0.005 (2)
C10	0.063 (3)	0.065 (3)	0.064 (3)	0.013 (2)	−0.003 (2)	0.016 (2)
C11	0.0279 (15)	0.0421 (19)	0.050 (2)	−0.0007 (13)	−0.0054 (14)	−0.0020 (16)
C12	0.0281 (16)	0.0480 (19)	0.064 (2)	−0.0051 (15)	0.0089 (15)	0.0036 (18)
C13	0.0302 (15)	0.0403 (18)	0.054 (2)	−0.0019 (14)	0.0116 (14)	0.0073 (15)
C14	0.0289 (15)	0.0274 (16)	0.0404 (17)	0.0013 (10)	0.0079 (13)	−0.0007 (12)
C15	0.0319 (16)	0.0445 (19)	0.0402 (18)	0.0028 (13)	0.0133 (14)	0.0112 (15)
C16	0.056 (2)	0.059 (3)	0.071 (3)	0.0042 (19)	0.003 (2)	0.028 (2)
C17	0.048 (2)	0.083 (3)	0.043 (2)	0.006 (2)	0.0075 (17)	−0.004 (2)

### Geometric parameters (Å, º)

**Table:**

Pd—N1	2.040 (3)	C7—C8	1.519 (5)
Pd—N2	2.072 (2)	C8—C9	1.510 (5)
Pd—Cl1	2.3055 (8)	C8—C10	1.536 (5)
Pd—Cl2	2.3160 (8)	C8—H8	0.9800
N1—C1	1.487 (4)	C9—H9A	0.9600
N1—C4	1.500 (4)	C9—H9B	0.9600
N1—H1	0.8600	C9—H9C	0.9600
N2—C6	1.463 (4)	C10—H10A	0.9600
N2—C5	1.497 (4)	C10—H10B	0.9600
N2—H2	0.8600	C10—H10C	0.9600
C1—C2	1.521 (5)	C11—C12	1.375 (5)
C1—H1A	0.9700	C11—H11	0.9300
C1—H1B	0.9700	C12—C13	1.369 (5)
C2—C3	1.507 (5)	C12—H12	0.9300
C2—H2A	0.9700	C13—C14	1.388 (4)
C2—H2B	0.9700	C13—H13	0.9300
C3—C4	1.539 (5)	C14—C15	1.524 (5)
C3—H3A	0.9700	C15—C17	1.524 (5)
C3—H3B	0.9700	C15—C16	1.535 (5)
C4—C5	1.510 (4)	C15—H15	0.9800
C4—H4	0.9800	C16—H16A	0.9600
C5—H5A	0.9700	C16—H16B	0.9600
C5—H5B	0.9700	C16—H16C	0.9600
C6—C7	1.399 (4)	C17—H17A	0.9600
C6—C14	1.410 (4)	C17—H17B	0.9600
C7—C11	1.392 (5)	C17—H17C	0.9600
			
N1—Pd—N2	83.98 (10)	C11—C7—C6	117.9 (3)
N1—Pd—Cl1	90.83 (7)	C11—C7—C8	119.3 (3)
N2—Pd—Cl1	174.45 (7)	C6—C7—C8	122.7 (3)
N1—Pd—Cl2	172.72 (7)	C9—C8—C7	110.5 (3)
N2—Pd—Cl2	92.27 (8)	C9—C8—C10	109.8 (3)
Cl1—Pd—Cl2	93.10 (3)	C7—C8—C10	113.6 (3)
C1—N1—C4	105.8 (2)	C9—C8—H8	107.5
C1—N1—Pd	119.3 (2)	C7—C8—H8	107.5
C4—N1—Pd	111.56 (18)	C10—C8—H8	107.5
C1—N1—H1	106.5	C8—C9—H9A	109.5
C4—N1—H1	106.5	C8—C9—H9B	109.5
Pd—N1—H1	106.5	H9A—C9—H9B	109.5
C6—N2—C5	111.0 (2)	C8—C9—H9C	109.5
C6—N2—Pd	121.74 (19)	H9A—C9—H9C	109.5
C5—N2—Pd	107.90 (18)	H9B—C9—H9C	109.5
C6—N2—H2	104.9	C8—C10—H10A	109.5
C5—N2—H2	104.9	C8—C10—H10B	109.5
Pd—N2—H2	104.9	H10A—C10—H10B	109.5
N1—C1—C2	102.5 (3)	C8—C10—H10C	109.5
N1—C1—H1A	111.3	H10A—C10—H10C	109.5
C2—C1—H1A	111.3	H10B—C10—H10C	109.5
N1—C1—H1B	111.3	C12—C11—C7	121.5 (3)
C2—C1—H1B	111.3	C12—C11—H11	119.3
H1A—C1—H1B	109.2	C7—C11—H11	119.3
C3—C2—C1	103.2 (3)	C13—C12—C11	119.4 (3)
C3—C2—H2A	111.1	C13—C12—H12	120.3
C1—C2—H2A	111.1	C11—C12—H12	120.3
C3—C2—H2B	111.1	C12—C13—C14	122.5 (3)
C1—C2—H2B	111.1	C12—C13—H13	118.8
H2A—C2—H2B	109.1	C14—C13—H13	118.8
C2—C3—C4	105.9 (3)	C13—C14—C6	117.1 (3)
C2—C3—H3A	110.6	C13—C14—C15	117.9 (3)
C4—C3—H3A	110.6	C6—C14—C15	125.0 (3)
C2—C3—H3B	110.6	C17—C15—C14	112.0 (3)
C4—C3—H3B	110.6	C17—C15—C16	111.6 (3)
H3A—C3—H3B	108.7	C14—C15—C16	110.0 (3)
N1—C4—C5	108.3 (2)	C17—C15—H15	107.7
N1—C4—C3	105.2 (3)	C14—C15—H15	107.7
C5—C4—C3	113.3 (3)	C16—C15—H15	107.7
N1—C4—H4	110.0	C15—C16—H16A	109.5
C5—C4—H4	110.0	C15—C16—H16B	109.5
C3—C4—H4	110.0	H16A—C16—H16B	109.5
N2—C5—C4	111.2 (2)	C15—C16—H16C	109.5
N2—C5—H5A	109.4	H16A—C16—H16C	109.5
C4—C5—H5A	109.4	H16B—C16—H16C	109.5
N2—C5—H5B	109.4	C15—C17—H17A	109.5
C4—C5—H5B	109.4	C15—C17—H17B	109.5
H5A—C5—H5B	108.0	H17A—C17—H17B	109.5
C7—C6—C14	121.6 (3)	C15—C17—H17C	109.5
C7—C6—N2	119.0 (3)	H17A—C17—H17C	109.5
C14—C6—N2	119.4 (3)	H17B—C17—H17C	109.5

### Hydrogen-bond geometry (Å, º)

**Table:**

*D*—H···*A*	*D*—H	H···*A*	*D*···*A*	*D*—H···*A*
N1—H1···Cl2^i^	0.86	2.47	3.283 (3)	158
N2—H2···Cl1^i^	0.86	2.68	3.410 (3)	144

Symmetry code: (i) −*x*+3/2, −*y*+3/2, −*z*+2.
